# Weight Loss and Decrease of Body Mass Index during Allogeneic Stem Cell Transplantation Are Common Events with Limited Clinical Impact

**DOI:** 10.1371/journal.pone.0145445

**Published:** 2015-12-18

**Authors:** Christina T. Rieger, Isabel Wischumerski, Christian Rust, Michael Fiegl

**Affiliations:** 1 Department of Internal Medicine III, Klinikum der Universität München, Munich, Germany; 2 Department of Internal Medicine I, Krankenhaus der Barmherzigen Brüder, Munich, Germany; University of Kentucky, UNITED STATES

## Abstract

**Purpose:**

Weight loss in cancer patients has been attributed with significant morbidity and mortality. During allogeneic stem cell transplantation (SCT), oral nutrition is often hampered and hence total parenteral nutrition (TPN) is necessary. We therefore investigated the course of weight during stem cell transplantation and the clinical consequences of weight change.

**Methods:**

180 consecutive patients who received allogeneic SCT between January 2010 and December 2011 at our center were analyzed for weight loss, laboratory and clinical parameters.

**Results:**

During SCT, a median decrease of 6.6% of body mass index (BMI) was observed for the whole population (from 25.3 at admission to 23.6 at discharge), and a 1.6fold increase of malnutrition despite use of TPN (28.3% to 45.0%). 55.6% of patients experienced a significant weight loss of ≥5% with a median decrease of 9.2% in BMI. Serum levels of albumin, total protein and cholesterol rapidly decreased during conditioning therapy. After a median of 2.4 years, the median BMI was still only 23.4 (not different from discharge). However, we did not observe a meaningful difference in side effects and survival between patients that did or did not lose weight.

**Conclusion:**

Weight loss is commonly observed during allogeneic SCT despite TPN, but the clinical consequences thereof seem limited: we observed no significant impact on patients with a decrease ≥ 5% in BMI on transplant outcome, side effects or survival.

## Introduction

Over the last decades, allogeneic stem cell transplantation (SCT) has become an important treatment option for malignant and non-malignant hematopoietic diseases [1]. However, the curative potential of this procedure is hampered by a clinically significant treatment-related morbidity and mortality. Transplant-related morbidity comprises of several problems, ranging from neutropenic infections, side effects of drugs and radiation, mucositis to (acute and chronic) graft versus host-disease (GvH-D). Besides therapy-related mortality, mostly due to infections, relapse remains one of the most common reasons for death after allogeneic SCT.

Several risk factors have been identified to estimate the individual risk for the patient; the most common being the HCT-CI [2]. Among the parameters utilized is the body mass index (BMI) prior to SCT, with a BMI >30 being prognostically unfavorable. Besides overweight/ obesity, underweight has been attributed with a negative effect on survival as well [3].

Underweight and weight loss is associated with decreased survival in patients with various malignant diseases. During allogeneic SCT, patients often suffer from insufficient oral intake and need supportive nutritional therapy. Reasons for this insufficient intake include, but are not limited to, nausea and vomitus due to conditioning therapy, mucositis following chemotherapy/ radiation or during neutropenia and also intestinal GvH-D, resulting in severe diarrhea and malabsorption. Usually, patients will receive total parenteral nutrition (TPN), and the European Group for Blood and Marrow Transplantation Society (EBMT) has made the following statement with regard to nutritional support during SCT: “Nutritional support is an integral part of the supportive care of patients receiving HSCT and the main tool remains TPN. It seems to be prudent to administer TPN to patients undergoing HSCT if they have severe mucositis or gastrointestinal manifestations of GvH-D, when a long period of insufficient oral intake is anticipated” [4].

A clear recommendation of routine TPN commencing at a fixed time point before clinical need, i.e. start of chemotherapy or transplantation, can however not been given, as the use of routine TPN remains controversial: Weisdorf et al. [5], in one (with 137 patients) of the few randomized trials addressing this topic, found a significant benefit for patients that received routine TPN in advance to and throughout the transplantation period, even though most of the patients that were not randomized in the routine TPN arm also received nutritional support including total parenteral nutrition later on. However, this trial includes pediatric patients and is almost 30 years old, hence is not fully representative for the modern allogeneic stem cell transplantation setting in adults, especially as nowadays reduced intensity conditioning instead of myeloablative regimens are more commonly used. In addition, TPN is associated with significant side effects, and later meta-analyses found no clear benefit for routine TPN, as the potential positive side effects are counteracted by complications–mainly blood stream infections due to central venous lines [6]. Therefore, oral nutrition is currently favored and TPN is regarded only a matter of last resort, and the standard being an individualized approach where patients receive TPN in case of insufficient oral uptake.

It was thus the aim of our study to investigate the effects of this strategy as defined by the EBMT on the weight and body mass index of patients undergoing allogeneic transplantation and the clinical consequences thereof in one single center.

## Patients and Methods

### Patient Cohort

All patients who underwent allogeneic stem cell transplantation for various malignant diseases between January 2010 and December 2011 at our Department of Internal Medicine III of the Klinikum der Universität München, Munich, Germany, were eligible for analysis. In total, during this period 180 consecutive transplantations were registered and the respective patient characteristics are shown in Table 1.

**Table 1:** Patients characteristics.

Demographics	n (range or %)
Median Age in years [range]	52 (20–74)
Female patients	80 (44.4%)
**Underlying disease**	
Acute myeloid leukemia	93 (51.7%)
Myelodysplastic Syndrome	11 (6.1%)
Acute lymphoblastic leukemia	23 (12.8%)
Non-Hodgkin’s Lymphoma/ Multiple Myeloma	26 (14.5%)
Other	27 (14.9%)
Transplantation in remission	94 (52.2%)
**Transplantation setting**	
HLA-identical (10/10)	141 (78.4%)
HLA mismatch	39 (21.6%)
Sibling donors (including haploidentical donors)	48 (26.7%)
Matched unrelated donor	84 (46.7%)
Sorror Score	3 (0–10)
**Stem cell source & quantity**	
Bone marrow	40 (22.2%)
Stem cells from peripheral blood	132 (73.3%)
Cord Blood	8 (4.4%)
Number of CD34+ cells per transplant	Median 7.4 x 10^6^ kg body weight [1.5–27.8]
**Conditioning regimen**	
Myeloablative conditioning^a^	25 (13.9%)
Reduced intensity conditioning^b^	155 (86.1%)

Epidemiological and clinical parameters of all patients transplanted in the observational period (n = 180)

^a^ e.g. 12 gy total body irradiation, busulfan 16 mg/ kg body weight

^b^ e.g. FLAMSA (fludarabine, cytarabine, amsacrin + 4 gy TBI or busulfan 8 mg), FBM (fludarabine, BCNU, melphalan), FC (fludarabine, cyclophosphamide)

**Fig 1:**
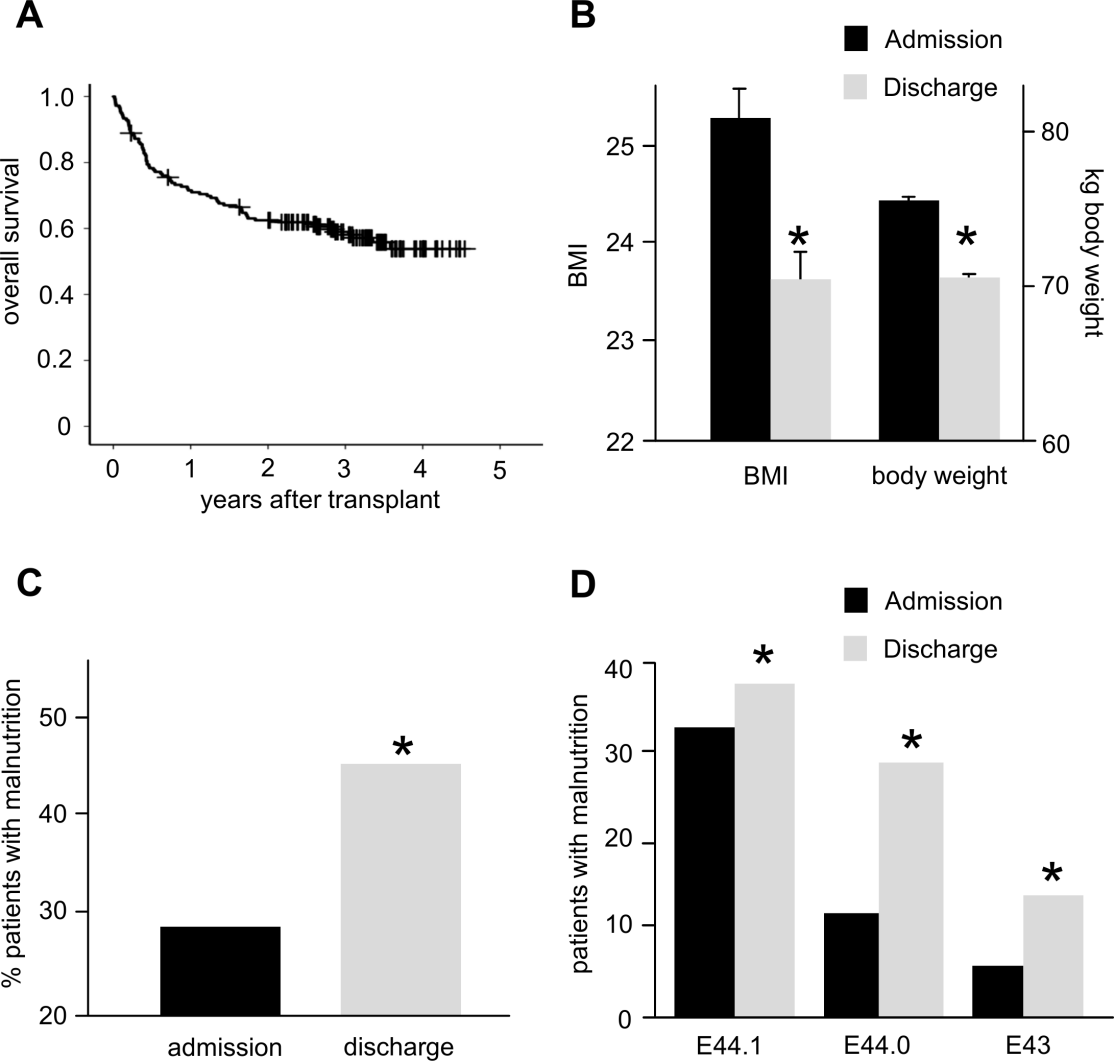
BMI change during allogeneic SCT. A: Overall survival of the patient cohort. The median survival is not reached after 5 years of follow up. B: Significant decrease of BMI and body weight at discharge as compared to admission. C: A significant increase of patients with malnutrition during SCT was observed. D: The percentage of patients with malnutrition increases during the period of allogenic SCT (severity of malnutrition was graded according to ICD-10).

Six patients died within 30 days after transplantation (3.3%), and median survival was not reached in the cohort after 5 years. The respective survival curve for the whole population is shown in Fig 1A.

### Data Collection

Data collection comprised of epidemiological, clinical and laboratory parameters to evaluate the impact of weight development during allogeneic SCT on these parameters. The data was routinely gathered at various time points depending on the respective parameter (e.g.: blood count and weight on a daily basis, serum albumin and total protein at least twice weekly or more often if clinically indicated), and documented for clinical purpose during the in-patient period and was later entered into a data base (Excel 2010, Microsoft Cooperation) for analysis. Data sets were fully anonymized for analysis. The study has been conducted according to the principles expressed in the Declaration of Helsinki and was approved by the Internal Review Board of the Department of Internal Medicine III, Klinikum der Universität München, Munich, Germany.

### Body Mass Index and Definitions for Malnutrition

BMI was calculated according to the formula [kg body weight / (body size)²] and grouped into different categories according to the WHO classification (<18.5: underweight, 18.5–25.0: normal weight, ≥25.0–30.0: overweight, >30.0 obesity).

For analysis of malnutrition, weight loss was categorized according to ICD-10: E43 (unspecified severe protein-energy malnutrition), E44.0 (moderate protein-energy malnutrition) and E44.1 (mild protein-energy malnutrition). Categorization followed the definitions by DIMDI (*Deutsches Institut für Medizinische Dokumentation und Information* [7]): E43: body weight was below 3 standard deviations of the age and sex-adjusted mean body weight of the normal population, E44.0: body weight was below 2 standard deviations of the age and sex-adjusted mean body weight of the normal population, E44.1: body weight was below 1 standard deviations of the age and sex-adjusted mean body weight of the normal population.

### Nutrition during Allogeneic Stem Cell Transplantation

Parenteral nutrition was not regulated by this study and followed the internal institution standards, adjusted on an individual basis. Generally, parenteral nutrition was administered via a central venous line and total parenteral nutrition (TPN) usually commenced around the time point of transplantation or earlier, depending on the ability of the patient to sufficiently take in food or nutrients orally (by judgement of the treating physician).

TPN comprised of 2000 mL commercial available mixture (Nutriflex Basal^®^, B. Braun Melsungen AG, Melsungen, Germany) supplemented with 250 mL of lipids (ClinOleic 20%^®^, Baxter Deutschland, Unterschleißheim, Germany) with additional supplementation of minerals and vitamins. Thus, TPN contained app. 1760 kcal daily (1260 kcal for Nutriflex^®^ and 500 kcal for lipids). TPN usually continued for 2–3 weeks during aplasia; and thereafter parenteral nutrition was subsequently tapered over 1–2 weeks on an individual basis until sufficient oral intake by the patient was possible. During the whole transplantation period, patients were offered and encouraged to consume food orally.

### Statistical Analysis

Statistical calculations were performed with SPSS Statistics^®^ 23 (IBM Deutschland GmbH, Ehningen, Germany). Statistical methods applied are given in the appropriate section of the text and comprised student’s t-test, the method of Kaplan-Meier and Persons X^2^ test. Results are given as median values with range, standard deviation or standard error of the mean. A p < 0.05 was considered statistically significant.

## Results

### BMI and Weight Change during Allogeneic Stem Cell Transplantation

The initial weight parameters of all 180 patients are given in Table 2. Median age was 52 years (20–74) and the median BMI 25.3 (17.3–47.8). This BMI compares favorably to the normal median BMI of the German population of 50–55 years in 2013, which was 26.4 [8]. The majority of patients (52.8%) had a BMI > 25, with 40.6% (n = 73) being overweight and 12.2% (n = 22) being obese. On the other side, 51 patients (28.3%) displayed signs of malnutrition at the time point of admission.

**Table 2:** Nutritional parameters of patients.

Weight (kg)	n (range or %)
All patients	75.4 (45.9–130.0)
Female patients	66.5 (45.9–130.0)
Male patients	81.0 (60.6–122.8)
**Body mass index**	
All patients	25.3 (17.3–47.8)
Female patients	23.9 (17.3–47.8)
Male patients	25.9 (17.3–37.0)
**Weight categories according to BMI**	
Underweight	5 (2.8%)
Normal weight	80 (44.4%)
Overweight	73 (40.6%)
Obesity	22 (12.2%)
**Malnutrition**	
E44.1	33 (18.3%)
E44.0	12 (6.7%)
E43.0	6 (3.3%)

Body mass index, weight and incidence of malnutrition of all patients at the beginning of the observational period; i.e. admission for allogeneic transplantation

At the time point of discharge, which was a median of 41 days after transplantation, the median BMI was 23.6%, 6.6% lower than at admission (p<0.01, Fig 1B). Accordingly, weight dropped from 75.4 kg to 70.1 kg (p<0.01, Fig 1B). This was found for both sexes (BMI: ♀ 23.9 vs. 22.8, p<0.01, ♂ 25.9 vs. 24.6, p<0.01, weight: ♀ 66.5 vs. 62.9 kg, p<0.01, ♂ 81.0 vs. 75.8 kg, p<0.01).

The amount of patients with malnutrition increased from 28.3% (n = 51) at admission to 45.0% (n = 81, Fig 1C). This increase however was not equally distributed between the different groups of malnutrition: while all groups significantly increased, the highest increment was observed in the group with moderate protein-energy malnutrition (1.2fold for E44.1, 2.4fold for E44.0 and 2.3fold for E43, Fig 1D).

### Patients at Risk for Weight Loss and Weight Development after SCT

**Fig 2:**
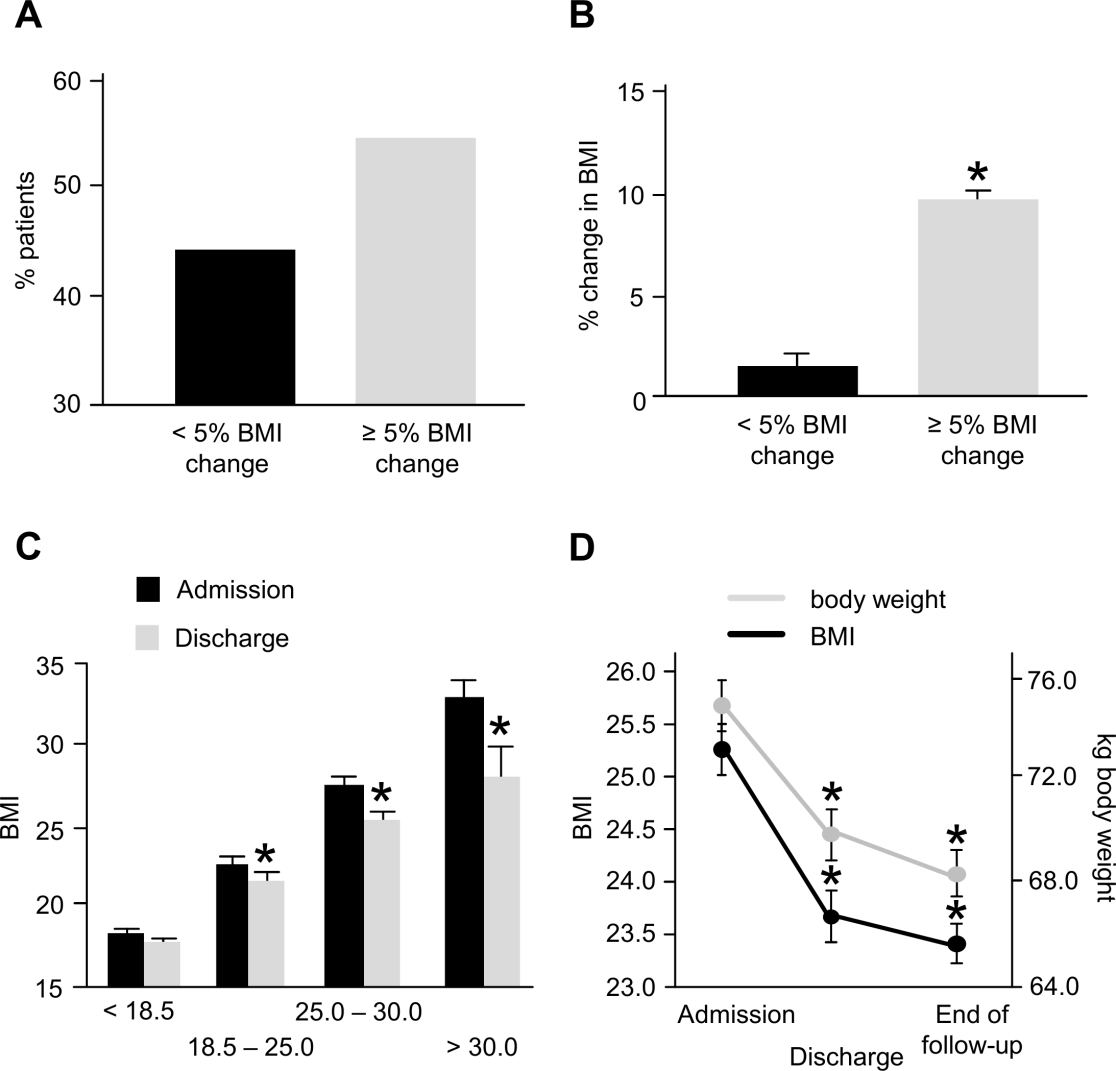
Distribution of weight loss and long-term follow-up. A: App. 40% of patients decreased < 5% of BMI (considered not significant), but the majority lost ≥ 5% of BMI during SCT. B: In the group of patients whose BMI decreased, the loss was significantly 6.1fold higher. C: Overweight and obese patients lost significantly more weight than normal or underweight patients. D: Both BMI and body weight stayed at the same low level at discharge even during long term follow-up.

After establishing a significant weight loss during allogeneic SCT, we next examined whether this weight loss was equally distributed. However, if a cut-off value of 5% loss of BMI was chosen (which defines a CTC I° toxicity [9]), 44.4% (n = 80) had < 5% BMI loss (with 8 gaining > 5% of BMI) and 55.6% (n = 100) lost ≥ 5% (Fig 2A). The median decrease in the group of patients that lost ≥ 5% was 9.2% but 1.5% in the group of patients that lost < 5% (Fig 2B).

As these data suggest an uneven distribution of BMI/ weight loss in the group of stem cell transplantation recipients, we aimed to identify patients at increased risk. Interestingly, when the different weight groups at admission according to WHO were analyzed, it became obvious that while all groups (except underweight) lost weight and decreased BMI, the loss was greatest in the group of initially obese patients. As shown in Fig 2C, underweight patients lost a non-significant 2.5% in BMI, but the decrease was 5.3% in the normal weight group, 6.2% in the overweight group and 10.2% in the obese group (each p < 0.01). Similar results were found for body weight change (underweight: 55.4 vs. 53.5 kg, n.s., normal weight: 66.9 vs. 63.2 kg, p < 0.01, overweight: 80.0 vs. 74.7 kg, p < 0.01, obese: 100.0 vs. 88.75 kg, p < 0.01).

We then investigated the development of BMI after the in-patient period for SCT (Fig 2D). We found that in 144 patients, in which a body weight was available at least 90 days after discharge, at the end of follow-up (median 873±361 days) the median BMI was 23.4 (±4.1) and median weight was 68.5 kg (±20.0 kg). Both values were significantly lower than at admission and as low as at the time point of discharge, more than a median of 2 years earlier.

### Change of Laboratory Parameters Associated with Metabolism

**Fig 3:**
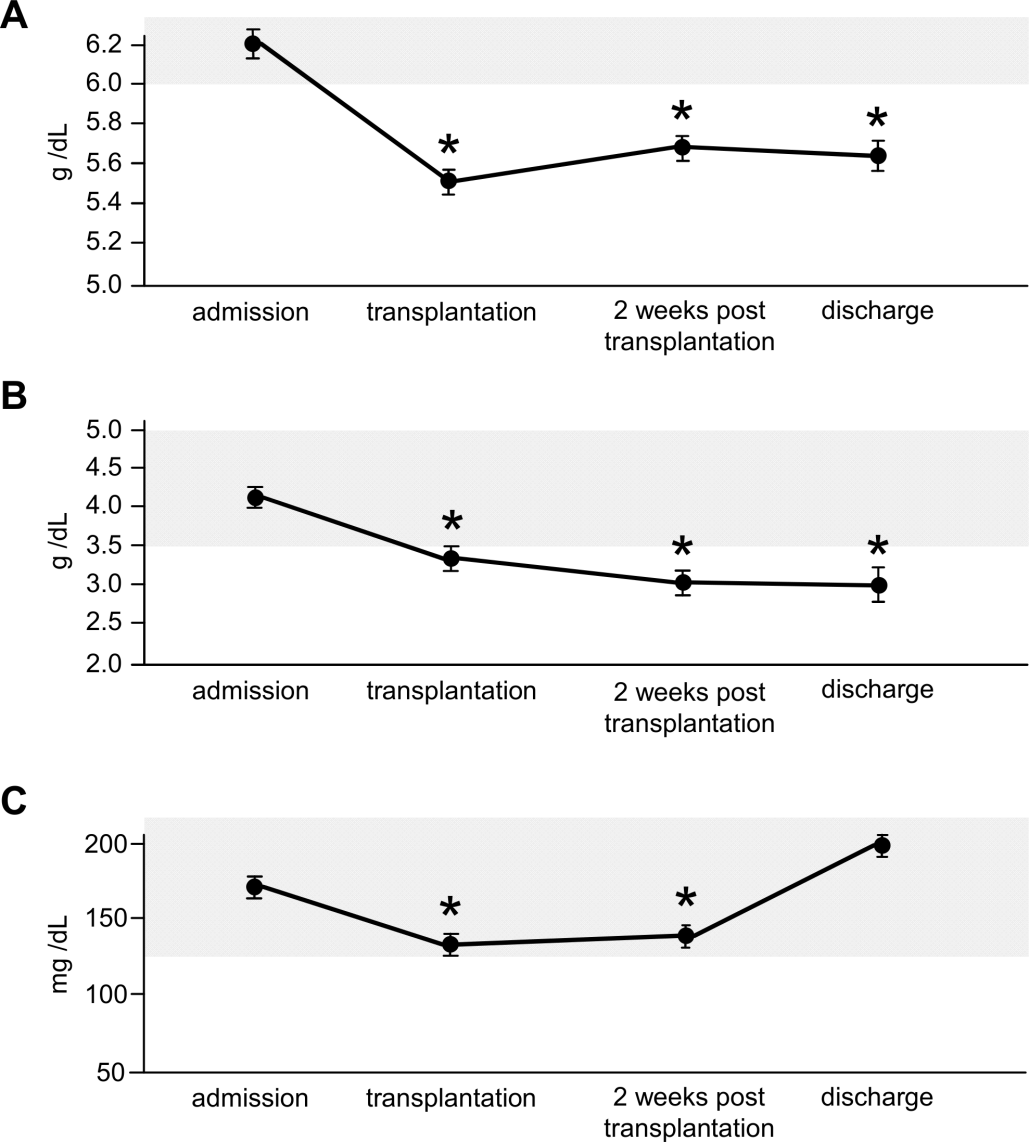
Serum levels of metabolic relevant parameters. A: Significant decrease of total serum protein at the time point of transplantation, remaining low despite TPE. B: Similar results for serum albumin. C: Significant decrease of cholesterol, with recovery to pre-transplant values at the time point of discharge. Grey area encompasses normal range.

Next, we were interested in serum parameters associated with metabolism and their development during allogeneic SCT. Total serum protein (Fig 3A) was within the normal range (NR) during admission (median 6.2 g/dL, NR 6.0–8.0 g/dL), but decreased significantly below the lower normal level to 5.5 g/dL at the time point of transplantation (decrease of 11.3%) and stayed at this low level during aplasia (5.7 g/dL) and at discharge (5.7 g/dL) despite TPN. Similar results were obtained for serum albumin (Fig 3B): at admission the median was 4.2 g/dL (NR 3.5–5.0 g/dL), but it significantly decreased to 3.4 g/dL at the time point of transplantation (19% decrease), staying low during aplasia (3.0 g/dL) and at discharge (3.3 g/dL). Cholesterol (Fig 3C) also significantly decreased early on (NR 120–240 mg/dL) from normal values at admission (median 168 mg/dL) to 141 mg/dL at transplantation (16% decrease), staying low during aplasia (147 mg/dL) but returned to initial values at discharge (200 mg/dL), never leaving the normal range.

### Clinical Consequences of Weight Loss during Allogeneic Stem Cell Transplantation

**Fig 4:**
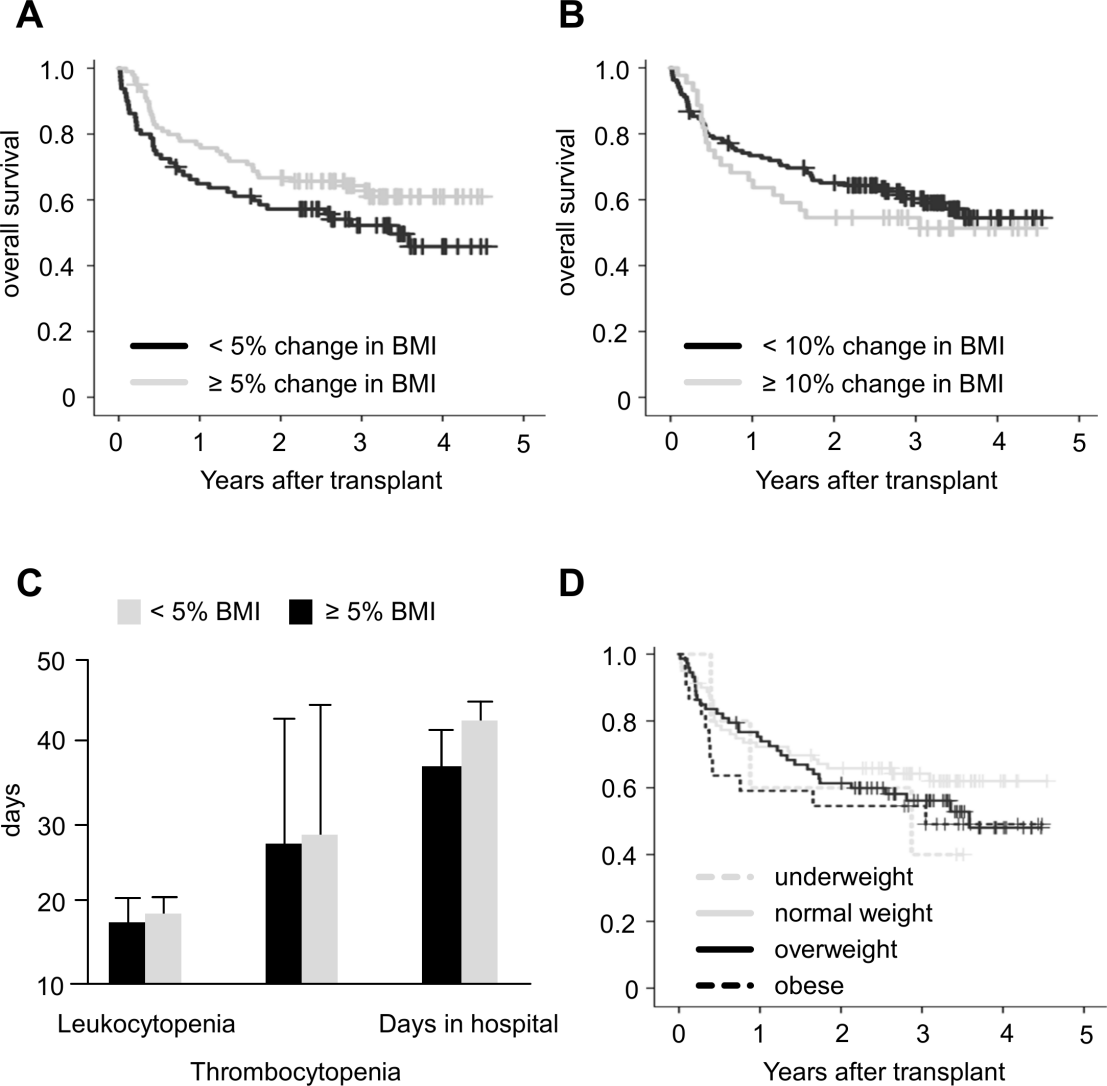
Clinical consequences of weight loss during stem cell transplantation. A: No difference in overall survival between patients that lose ≥ 5% of BMI during SCT B: Similar result for a BMI decrease of ≥ 10%. C: No significant difference for leukopenia, thrombocytopenia or days in hospital according to BMI decrease. D: No significant difference in overall survival according to weight at admission.

As weight loss in cancer patients has been attributed with relevant clinical consequences, we next investigated the effects of weight loss on the outcome of SCT patients. As the most important factor is overall survival (OS), we analyzed whether patients with a decrease of BMI ≥ 5% had an inferior outcome than patients without loss, but failed to observe any significant difference (median OS for patients without weight loss: 3.4 years, OS for patients with ≥ 5 weight loss: not reached, p = 0.085, Fig 4A). Similar results were obtained for the event free survival (EFS, data not shown). We also chose a 10% decrease of BMI as a threshold (n = 44 ≥ 10% decrease), but also observed no significant difference here (median OS in both groups not reached, p = 0.456, Fig 4B). Accordingly, no effects on EFS were observed (data not shown).

Next, we investigated the effect of weight loss on side effects of SCT. We did not observe a significant effect of BMI decrease of 5% on leukocytopenia (median 18.5 vs. 19.0 days), thrombocytopenia (median 28.0 vs. 28.5 days; if no recovery was observed, the last follow-up date was recorded as recovery date) or days in hospital after transplantation (median 38.5 vs. 42.0 days, Fig 4C). Accordingly, no significant differences were found for the amount of erythrocyte (14.0 vs. 16.0) and thrombocyte (16.0 vs. 17.5) transfusions. We also failed to observe an impact on the duration on leukocytopenia (18.5 vs. 19.0) or thrombocytopenia (28.0 vs. 29.0) if a cut-off of 10% loss was chosen, but there was a small, yet statistically significant effect on the duration of hospitalization (38.5 vs. 41.0 days, p<0.01).

Also, there was no significant differences concerning infections (82.5% vs. 75%, p = 0.28) or days with fever (mean 4.0 vs. 3.0, p = 0.33) between both groups. When patients were analyzed according to suspected fungal infection, there was also no difference between both groups (42.5% vs. 44.0%, p = 0.88), as was for mucositis, which should serve as a trigger for TPN and thus a possible cause for malnutrition (57.5% vs. 51.0%, p = 0.45). Accordingly, neither grade nor duration of mucositis differed significantly between both groups (mean I° in both groups, mean duration 10.2 days versus 12.3 days).

Concerning GvH-D, 139 patients (77.2%) suffered from acute GvH-D (21.1% I°, 20.6% II°, 30.6% III° and 5% IV°), but there was no significant difference whether patients had a decrease in BMI of ≥ 5% (all grades: 73.8% vs. 80.0%, p = 0.37; I°: 20.0% vs. 22.0%, II°: 22.5% vs. 19.0% III°: 26.3% vs. 34.0%, IV°: 5% vs. 5%; severe GvH-D [III-IV°]: 42.4% vs. 48.8%). Thirty-two patients (17.8%) suffered from chronic GvH-D (n = 24 limited, 8 extensive); 20.3% in the group without decrease in BMI and 16.0% of patients within the cohort that lost ≥ 5% of BMI suffered from chronic GvH-D (p = 0.56).

### Outcome of Over- and Underweight Patients

We finally analyzed the effects of the initial BMI on the outcome in our patient collective. While we observed a trend for inferior outcome regarding to OS for underweight and obese patients (as shown in Fig 4D), this difference was not statistically significant (mean OS: underweight 2.2 years, normal weight 3.1 years, overweight 3.0 years, obese 2.6 years). Similarly, no significant effect of admission BMI was observed on EFS (data not shown).

## Discussion

Patients undergoing allogeneic SCT often face nutritional problems. Early on, routine TPN was recommended based on one clinical trial, which showed superiority of routine TPN over a more individual approach [5], but this view has been challenged due to other trials that failed to confirm this finding or even showed negative effects of TPN [6]. Currently, there is no recommendation for routine TPN, and the suggestions given by the EBMT [4] or elsewhere [10] are rather vague: TPN is recommended in patients who have severe mucositis or gastrointestinal manifestations of graft versus host disease, preventing sufficient oral intake over a prolonged time period. It was our goal to investigate the results of this demand-oriented approach in one single center study.

Our study, with 180 patients one of the largest to address incidence and consequences of weight loss during allogeneic SCT, confirms previous findings of a significant decrease of BMI during allogeneic stem cell transplantation [11]: in our cohort the median decrease was 6.6% for all patients, but in fact more than half of the patients had a decrease of ≥ 5%, and these patients had a decrease of almost 10% during the in-patient period. It was interesting to see that this lower median BMI of 23.6 at discharge was maintained over a median follow-up of almost 2 years.

On the other hand, surprisingly we failed to observe a meaningful impact of weight loss ≥ 5% on survival parameters or side effects of SCT like infections, mucositis, etc. This finding was rather unexpected: weight loss/ malnutrition usually is associated with increased morbidity and mortality in cancer patients [12] or patients that undergo invasive procedures like surgery [13,14]. Also, weight loss has recently been attributed with non-relapse mortality in allogeneic SCT [15]. Of course, the main problem with investigations like these is that malnutrition/ underweight merely correlates with morbidity–i.e. somebody very ill is likely to lose weight or to be cachectic, but that does not necessarily mean that correcting the weight will correct the underlying morbidity. The reason why there were no significant clinical consequences of weight loss during the in-patient period in our study however might be rather simple: the mere fact of weight loss is obviously not so important but rather who is affected by it. Indeed, in our study, the patients that experienced the most weight loss/ decrease in BMI were the initially overweight and obese patients. And it is without question that overweight in patients that are treated in curative intention–and are cured in a considerable proportion–has the same negative effects as in otherwise healthy humans, like increased mortality for arteriosclerosis, diabetes and even cancer [16]. Hence, weight normalization in overweight patients might rather improve and not impair their survival. In fact, the study by Fuji et al. [15] reported a negative effect of weight loss in allogeneic SCT, but it comprised mostly of Asian patients and the proportion of overweight and obese patients was much smaller (18.6%) than in our study (52.8%). In addition, the majority of these patients received myeloablative conditioning (69.7%), which differed from our collective where most patients were treated with RIC. Clearly the type of induction does impact outcome but is also likely to influence factors like food intake, absorption of nutrients and metabolism.

The persistence of a decreased BMI at the end of follow-up is of course suggestive of a hampered caloric intake exceeding the transplantation period itself and/ or that the patients might suffer from chronic GvH-D or other chronic diseases that affect oral nutrition. In a longitudinal follow-up of the nutritional status it was shown that weight loss during the post-transplantation period was influenced by allogeneic SCT-associated complications [11]. However, the percentage of patients with (chronic) GvH-D was rather low in our collective, and of course the severely morbid patients or patients that already succumbed to relapse or transplantation-associated complications were no longer available for analysis (only the ambulatory setting was surveyed). Hence, given the fact that during SCT it is the group of overweight/obese patients that predominantly lose weight, this decreased weight/ BMI might in reality not be as harmful as it seems but instead corrects harmful condition of overweight.

The use of only weight and BMI is a drawback of our study, as it may not be a precise enough marker. More modern measurements for the nutritional status of patients have been applied in the setting of allogeneic SCT [17], and besides the mere nutritional status of the patient, nutrition may also influence the metabolic profile which in turn might impact immunoregulation [18] and also posttransplant complications [19]. However, given the fact that little attention has been paid to nutrition at all, a simple measurement like BMI that can be utilized easily might suffice at first. To improve the validity of BMI, we also added (routinely measured) serum parameters associated with metabolism to our analysis, and found the decrease in BMI mirrored in the course of total protein and albumin and to a lesser degree in cholesterol. The most interesting observation in this respect was the early decrease in all 3 parameters already at the time point of transplantation (app. 10–14 days after admission)–where the factors that should trigger TPN (severe mucositis or GvH-D preventing oral intake of nutrients) are virtually absent. This fact calls into question whether it is only the reduced oral intake of nutrients but not rather also a catabolic state induced by chemotherapy and/ or radiation.

While not germane to our study, we failed to observe any effects of BMI at admission on the outcome. This seems to contradict other published data, but is probably due to the fact that our study was not powered to detect this difference. One recent study included >12,000 patients for an analysis to detect a rather moderate effect of e.g. overweight/obesity on non-relapse mortality (HR 1.19) [3]. Also, there are other studies that have shown the opposite, i.e. a higher OS in overweight patients, at least in certain allogeneic SCT settings [20], or no effect on survival but only on the incidence of side effects [21].

While further investigations, e.g. interventional trials, are necessary, several conclusions can be drawn from our study: (I) while the weight loss during allogeneic SCT is evident, this might not be necessarily a bad thing, as mainly the overweight/ obese patients are affected. Hence, when judging weight loss after SCT, not only the amount of decrease matters but also in which patients it is observed. (II) With a decrease of BMI of >5% (i.e. I° CTC) in 50%, weight loss has to be defined as a common side effect of allogeneic SCT, occurring despite nutritional support including TPN. Hence, patients need to be informed before SCT and this side effect should be included as a typical complication that patients need to give their informed consent to, even if this is not considered a serious event by the treating physicians. (III) In case patients lose weight during SCT, they should be informed that this weight loss is possibly long lived and might persist for 2 years or longer. This could relieve pressure from both patients and physicians to take questionable measures to increase weight. (IV) Even though the weight loss is common and significant, it does not result in impaired survival, increased relapse rate or increased incidence of side effects. Also, there was no effect on OS and EFS, which is important information to put the patients mind at rest. Especially the last 3 points should have immediate impact in patient information.

## Supporting Information

S1 Dataset Table containing different BMI, transplant setting and clinical parameters for all patients included. (PDF)
