# Supplementary material for: Weight Loss and Decrease of Body Mass Index during Allogeneic Stem Cell Transplantation Are Common Events with Limited Clinical Impact
Source: PLoS One. 2015 Dec 18;10(12):e0145445. doi: 10.1371/journal.pone.0145445 (PMC4689501; doi:10.1371/journal.pone.0145445)
Supplement: S1 Dataset — (PDF) [file pone.0145445.s001.pdf]

1	2	3	4	5	6	7	8
17,3	17,2	2	21	321	321	0	1
17,3	17,8	2	15	144	144	0	1
17,5	16,8	1	17	27	1048	0	1
17,5	17,1	1	18	21	1281	1	1
18,2	16,3	1	22	320	1250	1	1
19,1	17,7	2	58	792	1049	1	1
19,1	18,0	2	16	32	1130	0	0
20,1	19,7	2	16	13	670	0	1
20,1	19,3	2	13	16	1206	1	1
20,1	19,6	1	24	28	828	1	0
20,2	22,6	2	17	190	608	0	1
20,3	20,7	2	15	26	1461	1	1
20,6	20,9	2	52	490	951	1	1
20,7	20,3	2	21	23	1219	1	1
20,8	19,6	2	16	22	1130	1	0
20,9	20,8	2	13	12	155	0	0
20,9	19,8	2	39	195	1040	1	0
20,9	17,5	2	12	39	1246	1	1
21,1	20,1	1	26	25	1037	1	1
21,2	23,2	2	33	33	33	0	0
21,2	20,3	2	12	30	1195	1	1
21,3	21,2	2	15	27	159	0	0
21,3	20,5	1	20	17	1085	1	1
21,3	21,8	2	15	19	965	1	1
21,5	20,4	2	13	13	1658	1	1
21,5	20,7	2	22	14	1336	1	1
21,7	20,9	2	21	99	99	0	1
21,7	21,9	1	27	107	1330	1	1
21,8	21,5	1	19	29	231	0	1
21,8	20,6	2	16	17	1174	1	1
21,9	21,6	2	18	36	190	0	1
22,0	19,6	2	14	118	1253	1	1
22,0	20,6	1	26	35	1267	1	1
22,2	16,7	2	23	17	349	0	1
22,2	20,9	2	13	12	1317	1	1
22,2	20,5	2	31	18	84	1	1
22,2	21,8	2	16	21	960	0	1
22,3	24,1	2	26	80	80	0	0
22,3	20,9	2	24	20	626	0	0
22,4	20,4	2	16	29	957	1	0
22,4	23,9	2	11	11	11	0	0
22,4	21,7	2	13	23	1369	1	1
22,4	20,6	1	56	75	1191	1	1
22,5	24,6	2	155	155	155	0	0
22,7	23,2	2	12	12	12	0	1
22,7	20,8	2	35	705	1354	1	1
22,8	21,0	2	17	32	1148	1	1
22,8	21,1	2	15	95	1012	1	1
23,0	23,6	2	17	33	1315	1	1
23,1	22,1	2	15	17	291	0	1
23,1	22,7	2	41	41	257	0	1
23,4	21,1	2	16	147	971	1	1
23,4	22,6	2	14	15	1461	1	1
23,4	20,9	2	17	28	137	0	0
23,5	23,5	2	14	28	1282	1	1
23,5	23,6	2	25	23	871	1	1
23,5	21,5	2	27	28	1180	1	1
23,6	19,5	2	17	15	952	1	1
23,6	25,5	2	9	9	9	0	0
23,6	23,0	2	36	960	960	1	0
23,6	22,7	2	23	19	1260	1	1
23,7	22,6	2	41	405	1022	1	1

Legend

1 BMI Admission
2 BMI Discharge
3 Conditioning regimen (myeloablative: 1, RIC: 2)
4 Neutropenia (days)
5 Thrombocytopenia (days)
6 Overall survival (days)
7 Alive (yes: 1)
8 any GvH-D (yes: 1)

23,7	22,3	2	8	7	127	0	1
23,8	21,5	2	72	72	72	0	1
23,8	20,5	1	21	28	1449	1	1
24,0	22,4	2	14	12	1147	1	1
24,0	18,2	1	23	23	498	0	1
24,1	23,0	2	19	12	491	0	1
24,1	24,6	2	33	75	595	1	1
24,2	22,4	2	15	18	1027	1	1
24,2	24,8	2	8	8	8	0	0
24,2	21,9	2	13	11	1520	1	1
24,2	24,0	2	11	14	1210	1	1
24,2	21,7	1	34	61	159	0	1
24,3	24,2	2	18	22	1469	1	0
24,4	21,8	2	26	31	155	0	0
24,5	24,3	1	22	16	1528	1	1
24,6	23,6	2	15	73	168	0	0
24,6	23,9	2	19	16	842	1	1
24,7	21,3	2	13	35	739	1	1
24,7	20,8	2	55	145	145	0	1
24,8	23,3	2	28	23	1048	1	1
24,9	22,9	2	28	56	914	1	0
24,9	22,7	2	16	83	950	1	1
24,9	25,6	2	22	17	901	1	0
25,0	24,2	2	14	105	1308	0	1
25,1	24,7	2	14	33	1266	1	1
25,2	23,4	2	20	23	1274	1	1
25,2	19,3	2	13	224	224	0	1
25,3	22,6	2	32	120	120	0	1
25,3	24,1	1	12	139	1588	1	1
25,4	23,6	2	14	11	794	1	1
25,5	22,9	2	45	62	1358	1	1
25,6	22,5	2	14	113	171	0	1
25,6	24,2	2	19	30	443	0	0
25,6	22,1	2	14	13	1587	1	1
25,6	24,2	2	29	700	848	1	1
25,7	24,2	1	31	76	76	0	1
25,7	23,3	1	19	36	1190	1	1
25,7	24,5	2	19	29	269	0	1
25,8	23,2	2	71	71	71	0	1
25,8	22,4	1	21	167	811	1	1
25,8	27,0	2	17	3	1632	1	1
25,9	26,0	2	14	77	77	0	1
25,9	22,5	2	14	18	1202	1	1
26,2	26,4	2	19	137	971	1	1
26,2	25,5	2	14	12	1218	1	0
26,2	23,1	1	16	94	976	1	1
26,3	23,2	2	14	118	1113	1	1
26,3	23,9	2	15	79	777	0	1
26,4	25,4	2	16	49	1474	1	1
26,4	25,4	2	16	10	258	1	1
26,4	25,2	2	27	17	931	0	0
26,5	22,7	2	23	22	1059	1	0
26,5	25,1	2	44	1026	1026	0	1
26,6	27,1	2	27	30	945	1	1
26,6	25,2	2	17	25	1118	1	0
26,6	24,9	2	15	10	919	1	0
26,9	26,4	2	6	6	6	0	0
26,9	22,8	1	15	21	1552	1	1
27,0	20,1	2	32	197	197	0	1
27,1	26,6	2	14	40	42	0	1
27,2	21,6	2	13	12	269	0	1
27,3	25,5	1	15	23	1244	1	1
27,3	25,0	2	17	31	487	0	0

27,4	24,9	2	23	32	1351	1	1
27,4	27,1	2	18	48	48	0	1
27,4	26,0	2	20	18	868	1	0
27,5	24,7	2	20	20	1429	1	1
27,5	27,4	2	34	42	1286	1	1
27,6	26,4	2	19	18	818	1	1
27,8	26,7	2	19	40	407	0	1
27,9	26,1	2	15	30	814	1	1
27,9	30,8	1	33	81	81	0	1
27,9	26,3	2	19	31	606	0	1
27,9	28,2	2	18	36	36	0	0
28,0	22,5	2	16	26	579	0	1
28,1	26,0	2	18	24	1464	1	1
28,1	25,7	2	16	79	1128	1	1
28,1	24,6	2	16	14	1421	1	1
28,2	26,0	2	12	62	62	0	1
28,2	26,7	2	14	19	631	0	1
28,2	25,8	2	13	12	96	0	1
28,4	26,6	2	13	12	1134	1	1
28,4	26,3	2	20	17	968	1	1
28,5	26,7	2	39	28	1209	1	0
28,5	26,5	2	25	38	1253	1	1
28,6	25,4	2	15	17	460	0	1
28,7	26,8	2	27	24	732	1	1
28,8	28,7	2	34	39	1076	1	1
29,0	28,8	2	74	74	74	0	1
29,0	28,7	2	51	58	1224	0	1
29,1	28,0	2	26	29	353	0	1
29,1	25,0	2	19	27	369	0	1
29,2	28,3	2	30	26	635	0	1
29,5	29,6	2	15	28	893	1	1
29,6	28,2	2	29	35	522	0	1
29,7	25,2	2	14	35	1147	1	1
30,2	25,6	2	71	33	101	0	1
30,5	26,4	2	20	45	1033	1	1
30,6	30,4	2	24	22	1162	1	1
30,7	28,4	1	23	20	1618	1	1
31,1	30,2	2	19	35	1281	1	1
31,1	28,5	2	31	38	276	0	1
31,2	27,2	2	17	23	136	0	1
31,4	26,5	1	19	23	1112	1	1
31,6	28,2	2	32	32	32	0	0
31,8	28,7	2	17	15	1314	1	1
31,8	28,6	2	17	12	1636	1	1
32,1	28,2	1	32	204	1198	1	1
33,3	28,6	2	19	20	1016	1	1
33,4	31,4	2	17	28	140	0	0
33,8	29,1	2	20	16	120	0	0
34,1	30,5	2	19	21	1112	0	1
34,2	38,3	2	45	45	45	0	0
34,4	28,8	2	35	41	604	0	0
35,3	32,2	2	19	151	151	0	1
36,0	30,7	2	23	28	1527	1	1
37,0	33,3	2	14	12	1260	1	1
47,8	49,9	2	13	28	28	0	1
